# Association between cumulative average BMI and cognitive decline: a 24-year cohort study

**DOI:** 10.1007/s00415-026-13696-2

**Published:** 2026-02-27

**Authors:** Qianhui Xu, Meng Hsuan Sung, Zhuo Chen, Janani Rajbhandari-Thapa, Grace Bagwell Adams, M. Mahmud Khan, Ye Shen, Xiao Song, Xia Song, Suhang Song

**Affiliations:** 1 Graduate School of Art and Science, New York University, New York, NY 10012 USA; 2 Department of Epidemiology and Biostatistics, College of Public Health, University of Georgia, Athens, GA 30602 USA; 3 Department of Health Policy and Management, College of Public Health, University of Georgia, Athens, GA 30602 USA; 4 School of Economics, Faculty of Humanities and Social Sciences, University of Nottingham Ningbo China, Ningbo, 315100 China; 5 Department of Traditional Chinese Medicine and Rehabilitation, The Fifth People’s Hospital of Jinan, Jinan, 250000 Shandong China

**Keywords:** Cognitive decline, Cumulative average body mass index (caBMI), Longitudinal cohort study, Aging population, Health and Retirement Study (HRS), Neuroepidemiology

## Abstract

**Background:**

High Body Mass Index (BMI) is linked to poor cognitive performance, yet limited studies have examined the long-term impact of cumulative average BMI (caBMI) on cognitive health. This study explores the association between caBMI and cognitive decline and identifies the critical time window during which caBMI has the strongest association with cognitive decline.

**Methods:**

Data were obtained from the Health and Retirement Study (1996–2020). Global cognition was assessed using a standardized composite score of memory and executive function. Cumulative average BMI (caBMI) was computed as the mean of the area under the BMI curve over the follow-up period. Linear mixed models assessed the associations between caBMI and cognitive decline, adjusting for sociodemographic characteristics, lifestyle, and health conditions.

**Results:**

Among 8252 cognitively healthy participants (mean age 59.0 [Standard Deviation (SD) = 5.6] years, 58.2% women, mean follow-up 17.5 (SD = 7.0) years), a 100-unit increase in caBMI was significantly associated with faster cognitive decline: global cognition (−0.0030 SD/year, 95% CI: −0.0036, −0.0024), executive function (−0.0028 SD/year, 95% CI: −0.0034, −0.0021), and memory (−0.0017 SD/year, 95% CI: −0.0023, −0.0011) (all *p* < 0.001). Year eight was observed as the time point at which caBMI showed the strongest association with declines in global cognition, memory, and executive function. Subgroup analyses revealed that caBMI was associated with greater cognitive decline in older adults (≥ 65 years), compared to the younger adults (50–65 years).

**Conclusions:**

caBMI was significantly associated with cognitive decline, with the strongest association observed 8 years later. These findings highlight the importance of long-term weight management and BMI monitoring in cognitive health assessments.

**Supplementary Information:**

The online version contains supplementary material available at 10.1007/s00415-026-13696-2.

## Introduction

Alzheimer’s disease-related dementia (ADRD), the most prevalent form of dementia, has emerged as a leading cause of death in the US, with 1 in 3 older adults dying with ADRD in 2023 [1]. Currently, nearly 6.7 million US adults aged 65 or older are estimated to be living with dementia, and this number could rise to nearly 12 million by 2040 [2]. This alarming trend highlights a significant aging crisis, underscoring the urgent need for strategies to safeguard the cognitive health of the elderly. While no curative treatments for ADRD currently exist, ongoing efforts to identify and address modifiable risk factors are crucial for preventing or delaying cognitive decline and the onset of dementia.

Higher Body Mass Index (BMI) has been reported to be one of the modifiable risk factors associated with poorer cognitive performance and accelerated cognitive decline [3]. Although the underlying mechanisms remain unclear, prior studies have suggested that higher BMI may influence cognitive aging through alterations in brain structure and function, potentially mediated by neuroinflammatory processes and cerebrovascular dysregulation [4, 5]. Such brain-related alterations have been linked to impairments in cognitive processes, including memory and executive function [6–8]. In addition, elevated BMI may also modify the strength of the association between brain-related changes and cognitive outcomes. This modification may reflect broader systemic metabolic dysregulation associated with adiposity, including alterations in gut microbiome-derived metabolites [9, 10].

The role of longitudinal changes in BMI in cognitive decline remains unclear, with inconsistent evidence [4, 11–15]. While some studies have suggested no significant association between BMI changes and cognitive decline, others have reported that individuals with a sustained high BMI at different time points tend to exhibit worse cognitive performance later in life [16, 17]. These findings highlighted the limitations of relying solely on single-point BMI measurements (e.g., baseline BMI), which may fail to capture BMI changes over time. Even studies examining BMI changes typically calculated them as the difference between two single time points over a short time interval, failing to account for the dynamic and cumulative nature of BMI over extended periods [18, 19]. An alternative approach to relying on single-point or the difference in two single-point BMI measurements is to integrate BMI values across multiple time points, known as a cumulative average BMI (caBMI). A caBMI analysis offers a more comprehensive metric to assess the long-term accumulated burden of high BMI, which may better describe its role in cognitive decline. In addition, to date, no studies have identified the specific time periods during which BMI has the greatest association with subsequent cognitive decline. Analyzing the caBMI in a longitudinal cohort study allows for the identification of these critical windows, which could shed light on the relationship between BMI trajectory and cognitive decline. These findings may help inform when weight management strategies are most relevant in the context of cognitive aging.

To address these gaps, this study aimed to (1) investigate the longitudinal association between long-term caBMI and subsequent cognitive decline and (2) examine the critical time period during which caBMI exhibits the strongest association with cognitive decline.

## Methods

### Data source and study population

Data were obtained from the 24-year cohort Health and Retirement Study (HRS), spanning from Wave 3 (1996) to Wave 15 (2020). HRS is a nationally representative longitudinal study conducted biennially, involving individuals aged 50 years and older [20]. Information regarding the objectives, design, and methodologies of the cohorts is available in other studies [21]. All HRS participants provided verbal informed consent for their participation in the study; HRS data collection procedures were approved by the National Institute on Aging and Institutional Review Board at the University of Michigan (HUM00061128) [20]. All variables used in the analysis are publicly available. The analytic study was reviewed by the Institutional Review Board of the University of Georgia and determined as non-human research (#PROJECT00008358).

**Fig. 1:**
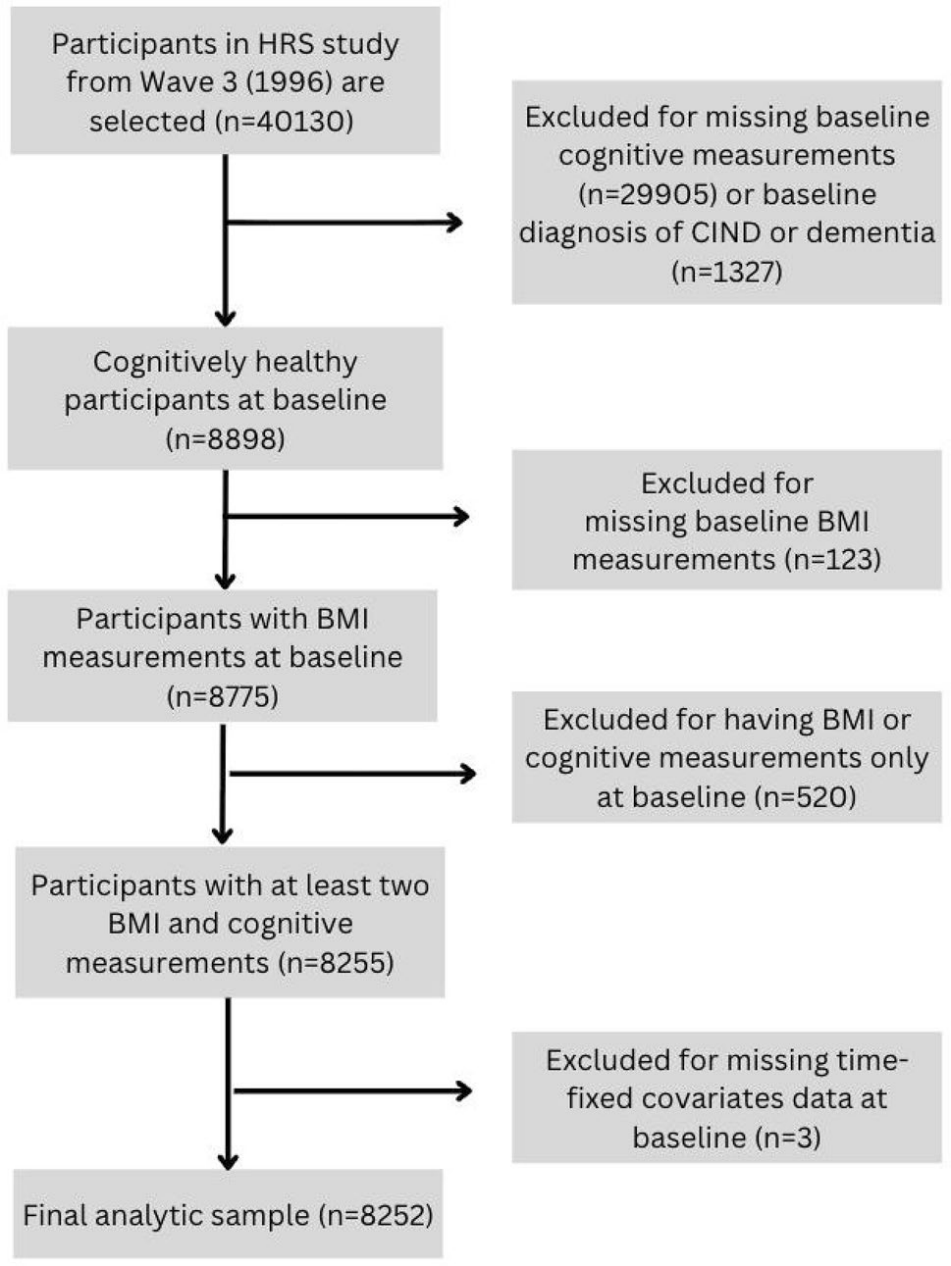
Flowchart of participants selection. Abbreviation: HRS: Health and Retirement Study; CIND: Cognitive impairment with no dementia; BMI: Body Mass Index

This study began with a total sample of 40,130 participants from Wave 3 (1996). We excluded participants who (1) did not have baseline cognitive measurements (*n* = 29,905), (2) were diagnosed as cognitively impaired but not demented (CIND) or demented at baseline (*n* = 1327), (3) lacked data for BMI at baseline (*n* = 123), (4) had only baseline measurement for either BMI or cognitive function (*n* = 520), and (5) had missing values on time-fixed covariates at baseline (*n* = 3). The final analytic sample consisted of 8252 participants. A flowchart detailing the selection process is shown in Fig. 1.

### Cumulative BMI measurement

BMI was calculated as weight/height^2^ using self-reported height (m) and weight (kg) [22]. Cumulative BMI was calculated using the Area Under the Curve (AUC) method, which plotted BMI measurements over time [23, 24]. The trapezoidal rule was used to determine the area under the curve, representing long-term exposure to BMI levels [23, 25]. Cumulative average BMI (caBMI) was calculated as the mean of cBMI values recorded across the follow-up period in years for each participant, updated at each wave interval. Detailed information on the calculation of caBMI is provided in Supplementary Fig. 1.

### Assessment of cognitive function

The outcome variables included three cognitive measures: two cognitive domains (i.e., memory and executive function) and global cognition. Memory domain was evaluated via immediate word recall (score range: 0–10) and delayed word recall (score range: 0–10) tests. Executive function was assessed through serial 7 subtraction (score range: 0–5) and backward counting (score range: 0–2) tests [26, 27]. A global cognitive score (score range: 0–27) was computed by summing the raw scores from memory and executive function. Higher scores indicated better cognitive performance [26, 28]. Cognitive outcomes were expressed as standardized Z-scores (SD units) to facilitate comparability across cognitive domains and across assessment waves in longitudinal analyses [29, 30]. Z-scores for each of the two cognitive domains and global cognition were calculated by subtracting the mean score at baseline from each individual data point in each wave and then dividing by the baseline standard deviation (SD) [31]. Cognitive decline rate was calculated as the change in standardized cognitive Z-scores from baseline to the follow-up time point, divided by the total follow-up duration for each participant.

The analysis accounted for both time-fixed and time-varying covariates. Time-fixed covariates included demographic factors measured at baseline (1996): age, gender (men or women), race and ethnicity (non-Hispanic White, non-Hispanic Black, non-Hispanic Other, or Hispanic), and educational attainment (less than high school, General Educational Development (GED), high school graduate, some college, or college and above). Time-varying covariates included depression status (yes or no), smoking status (never smoked, ever smoked, or current smoker), insurance status (insured or uninsured), employment status (employed, unemployed, retired, disabled, or not in the labor force), and the number of chronic diseases. The number of chronic diseases, ranging from 0 to 6, represents the summation of binary indicators for the following conditions: (1) hypertension or high blood pressure, (2) high blood sugar or diabetes, (3) cancer or any malignant tumor excluding skin cancer, (4) chronic lung disease excluding asthma, such as chronic bronchitis or emphysema, (5) heart attack, coronary heart disease, congestive heart failure, angina, or other heart conditions, and (6) transient ischemic attack or stroke.

### Statistical analysis

We conducted descriptive statistical analyses to summarize baseline characteristics. Continuous variables were reported as means with SD, while categorical variables were presented as frequencies and percentages. Statistical significance was set at the 5% level (*p* < 0.05) with two-tailed tests. All analyses were conducted using R Studio (Version 2024.04).

To examine the longitudinal association between caBMI and cognitive decline rate, we applied linear mixed models, which incorporated random intercepts at the participant level to account for baseline differences and individual variations. The models included an interaction term between caBMI and time, where time was measured as the number of years elapsed since the baseline year (1996) when caBMI was calculated, with a negative coefficient of the interaction term interpreted as indicative of a faster decline rate in cognition while holding time constant [32, 33].

We employed three models to sequentially add sets of characteristics, accounting for both non-modifiable and modifiable factors. Model 1 regressed caBMI on cognitive decline rate adjusted for demographic characteristics, including age, gender, and race/ethnicity to account for non-modifiable characteristics; Model 2 additionally adjusted for socioeconomic status (SES) (i.e., educational attainment, insurance, and employment status) to reflect modifiable factors; and Model 3 was a fully adjusted model with additionally accounting for lifestyle and health conditions (i.e., smoking, depression, and number of chronic diseases).

To determine the time period during which caBMI has the strongest association with cognitive decline, we applied a linear mixed model to analyze lag years across intervals ranging from 2 to 16 years. Lag years refer to the temporal delay between the caBMI measurement and its subsequent estimation of cognitive decline rate. At each exposure period, caBMI was recalculated using BMI measurements accumulated from baseline up to the end of that exposure period. Cognitive outcomes were then aligned to occur at specific lagged time points (2–16 years) following the exposure period. BMI measurements obtained after the exposure period, including those within the lag window, were not included in the caBMI calculation. For example, lag year two refers to the time interval in which the cognitive decline is assessed two years after the caBMI’s measurement. To better understand the influence of caBMI on cognitive decline across population groups, we further conducted stratification analyses by age, gender, and educational attainment. Sensitivity analyses were performed by excluding participants with chronic diseases at baseline. Inverse probability weighting (IPW)-based sensitivity analyses were also conducted to evaluate the potential impact of selective survival and loss to follow-up. All stratified and sensitivity analyses were conducted using the 8-year lag, which was selected as the reference time window.

## Results

### Study population characteristics

Table 1 presents the participants’ baseline characteristics. At baseline, the study sample had an average age of 59.0 years (SD = 5.6), with women comprising 58.2% of the cohort. The racial/ethnic composition was predominantly non-Hispanic White (78.7%). Most participants were either retired (33.9%) or not in the labor force (52.2%). Additionally, 39.5% of participants were never smokers, while 39.5% had ever smoked in the past. The baseline mean score on global cognition was 17.7 (SD = 3.3), with a mean memory score of 11.8 (SD = 2.9) and a mean executive function score of 5.9 (SD = 1.4). The follow-up duration was 17.5 (SD = 7.0) years on average. The mean BMI was 27.3 (SD = 5.1), while the mean caBMI was 27.7 (SD = 5.1).

**Table 1:** Baseline characteristics of participants

Variables	Characteristics
Global cognition (mean [SD])	17.7 (3.3)
Memory (mean [SD])	11.8 (2.9)
Executive function (mean [SD])	5.9 (1.4)
Age (mean [SD] in years)	59.0 (5.6)
Gender (*n* [%])	
Men	3446 (41.8)
Women	4806 (58.2)
Race/ethnicity (*n* [%])	
Non-Hispanic White	6492 (78.7)
Non-Hispanic Black	1024 (12.4)
Non-Hispanic Other	146 (1.8)
Hispanic	590 (7.2)
Educational attainment (*n* [%])	
Lower Than High-school	1548 (18.8)
GED	418 (5.1)
High-school graduate	2817 (34.1)
Some college	1796 (21.8)
College and above	1673 (20.3)
Employment status (*n* [%])	
Employed	726 (8.8)
Unemployed	166 (2.0)
Retired	2796 (33.9)
Disabled	258 (3.1)
Not in labor force	4306 (52.2)
Insurance (*n* [%])	
Yes	7436 (90.6)
No	773 (9.4)
Smoking (*n* [%])	
Never smoked	3111 (39.5)
Ever smoked	3117 (39.5)
Current smoker	1659 (21.0)
Depression (*n* [%])	
Yes	7254 (87.9)
No	995 (12.1)
BMI (mean [SD])	27.3 (5.1)
Number of chronic diseases (mean [SD])	0.8 (0.9)
Follow up duration years (mean [SD])	17.5 (7.0)

*SD* standard deviation, *BMI* Body Mass Index, *GED* General Educational Development

### Association between caBMI and cognitive decline

Table 2 presents the results of progressively adjusted models evaluating the association between caBMI and global cognitive decline rate, using an 8-year lag as an example. The magnitude of the association between caBMI and cognitive decline was consistent across all three models, sequentially adjusting for non-modifiable and modifiable factors (Table 2). Specifically, an increase of 100-unit caBMI was associated with an accelerated decline rate of 0.0030 SD/year in global cognition (−0.0030; 95% CI: −0.0036, −0.0024; *p* < 0.001). A statistically significant association was observed in declines in memory and executive function, with a faster decline rate of 0.0017 SD/year (95% CI: −0.0023, −0.0011; *p* < 0.001) and 0.0028 SD/year (95% CI: −0.0034, −0.0021; *p* < 0.001) with an increase in 100-unit caBMI, respectively (Supplementary Tables 2 and 3).

**Table 2:** The association between cumulative average BMI and global cognitive decline

	Model 1		Model 2		Model 3	
	Coeff. (95% CI)	*p* value	Coeff. (95% CI)	*p* value	Coeff. (95% CI)	*p* value
caBMI	−0.0021 (−0.0366, 0.0323)	0.904	0.0113 (−0.0232, 0.0459)	0.521	0.0142 (−0.0209, 0.0494)	0.428
caBMI*Time	−0.0030 (−0.0035, −0.0025)	< 0.001	−0.0032 (−0.0038, −0.0026)	< 0.001	−0.0030 (−0.0036, −0.0024)	< 0.001

Note: Model 1 includes demographic covariates (age, gender, and race/ethnicity). Model 2 additionally includes socioeconomic status (educational attainment, employment status, and insurance status). Model 3 further includes lifestyle and health conditions (smoking, depression, and number of chronic diseases)

*Coeff.* Coefficient, *caBMI* Cumulative Average Body Mass Index

In the sensitivity analyses, excluding individuals with chronic diseases at baseline, the associations between caBMI and cognitive decline remained consistent (Supplementary Table 4). Similarly, inverse probability weighting (IPW)-based sensitivity analyses yielded results comparable to the primary findings across all three cognitive domains (Supplementary Table 5).

### Identification of the critical period

Table 3 presents the magnitude of the association between caBMI and subsequent cognitive decline rates over the study period. The number of participants gradually decreased with a longer lag length, due to the structural requirement of longer survival and continued follow-up. Lag year eight was identified as the critical period during which caBMI showed the strongest association with cognitive decline rate. Specifically, the association between caBMI and global cognitive decline was consistently negative across lag years, with the strongest association observed at year 8 (−0.0030; 95% CI: −0.0036, −0.0024; *p* < 0.001). With 100-unit caBMI increase, global cognitive decline rates accelerated from 0.0009 SD/year at year 2 (95% CI: −0.0016, −0.0002; *p* = 0.015) to 0.0022 SD/year at year 4 (95% CI: −0.0028, −0.0016; *p* < 0.001), and 0.0018 SD/year at year 6 (95% CI: −0.0024, −0.0012; *p* < 0.001). After peaking at year 8, the decline rates gradually stabilized, reducing to 0.0028 SD/year at year 10 (95% CI: −0.0034, −0.0021; *p* < 0.001) to 0.0016 SD/year at year 16 (95% CI: −0.0028, −0.0004; *p* = 0.010). Executive function showed a trajectory paralleling that of global cognition, with significant negative associations observed across lag years 4–12 and year 16 whereas memory exhibited a generally similar pattern with significant associations spanning lag years 4–10. Across all three cognitive assessments, year 8 emerged as the time point at which the strongest association between caBMI and cognitive decline was observed, suggesting a potentially informative window during which weight management strategies may have greater relevance for slowing cognitive decline.

**Table 3:** The association between cumulative average BMI and subsequent cognitive decline in the following 2–16 years

	Global cognition Coeff. (95%CI) *p* value	Memory Coeff. (95%CI) *p* value	Executive function Coeff. (95%CI) *p* value
2 years (*N* = 7635)	−0.0009 (−0.0016, −0.0002) 0.015	−0.0006 (−0.0013, 0.0001) 0.113	−0.0005 (−0.0012, 0.0002) 0.149
4 years (*N* = 7190)	−0.0022 (−0.0028, −0.0016) < 0.001	−0.0013 (−0.0019, −0.0006) 0.001	−0.0020 (−0.0026, −0.0014) < 0.001
6 years (*N* = 6898)	−0.0018 (−0.0024, −0.0012) < 0.001	−0.0008 (−0.0014, −0.0002) 0.014	−0.0021 (−0.0027, −0.0015) < 0.001
8 years (*N* = 6592)	−0.0030 (−0.0036, −0.0024) < 0.001	−0.0017 (−0.0023, −0.0011) < 0.001	−0.0028 (−0.0034, −0.0021) < 0.001
10 years (*N* = 6188)	−0.0028 (−0.0034, −0.0021) < 0.001	−0.0016 (−0.0022, −0.0009) < 0.001	−0.0022 (−0.0028, −0.0015) < 0.001
12 years (*N* = 5673)	−0.0014 (−0.0022, −0.0007) < 0.001	−0.0005 (−0.0013, 0.0002) 0.168	−0.0009 (−0.0016, −0.0002) 0.019
14 years (*N* = 5250)	−0.0012 (−0.0022, −0.0003) 0.009	−0.0003 (−0.0013, 0.0006) 0.465	−0.0004 (−0.0013, 0.0005) 0.353
16 years (*N* = 4673)	−0.0016 (−0.0028, −0.0004) 0.010	0.0003 (−0.0009, 0.0015) 0.640	−0.0018 (−0.0030, −0.0006) 0.003

*Coeff.* Coefficient, *BMI* Body Mass Index

### Subgroup analyses

**Fig. 2:**
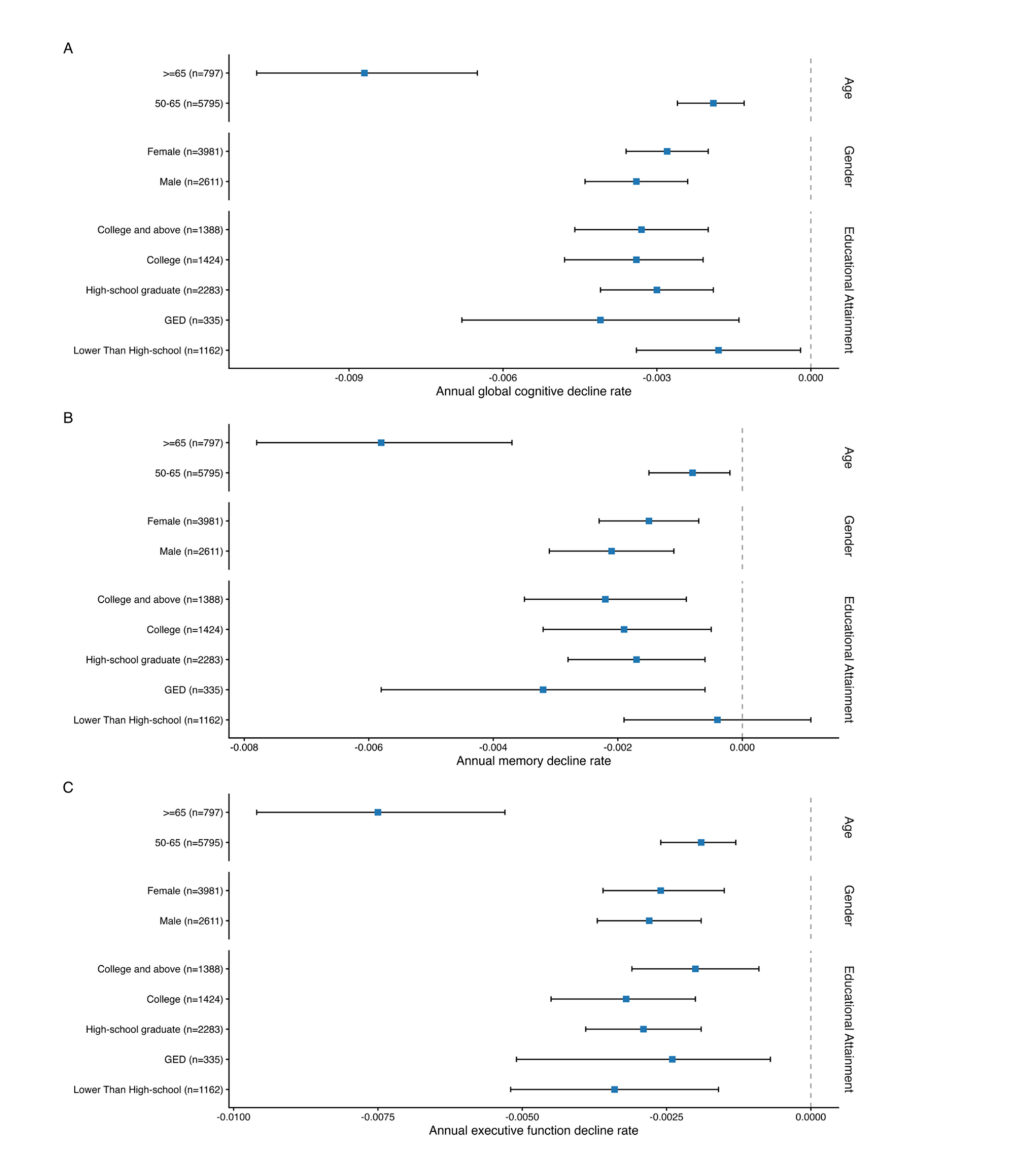
The association of cumulative average BMI with declines in global cognition (**A**), memory (**B**), and executive function (**C**) by age, gender, and educational attainment

After stratifying participants into subgroups, this study revealed statistically significant negative association betweencaBMI and global cognitive decline rate across each age, gender, and educational attainment group (Fig. 2). Among participants aged ≥ 65 years, an increase of 100-unit caBMI was linked to an accelerated global cognitive decline rate of 0.0088 SD/year (95% CI: −0.0109, −0.0067; *p* < 0.001). This negative relationship was significantly stronger in magnitude than in the younger group (50–65 years, −0.0019 SD/year; 95% CI: −0.0026, −0.0013; *p* < 0.001). Besides, the associations between increased caBMI and faster global cognitive decline were relatively consistent across gender and educational attainment, with similar decline rates observed for both men and women, as well as across all educational attainments. Similar trends were also observed in memory and executive function domains, highlighting the greater susceptibility of older adults to the association between caBMI and cognitive decline.

## Discussion

This population-based cohort study with average BMI of 27.3 (SD = 5.1) over a follow-up period of 17.5 years (SD = 7.0) on average, which innovatively demonstrated that higher caBMI exposure was associated with subsequent cognitive decline, with the greatest association being observed in the following eighth year. By measuring longitudinal BMI exposure, our findings highlight the importance of sustained BMI management and its role in long-term prevention strategies for cognitive decline. The association between higher caBMI and faster declines in global cognition, memory, and executive function suggests that long-term BMI patterns should be considered in cognitive risk assessments by clinicians.

As a modifiable risk factor, BMI represents a crucial target for interventions designed to reduce obesity-associated cognitive decline, although some studies observed that accumulation of cognitive reserve may help buffer against the negative effects of high BMI on cognitive decline in later life [34, 35]. Our study aligns with previous research demonstrating that higher BMI is associated with cross-sectional poorer cognitive function [36] and longitudinal cognitive decline [37], underscoring the role of cBMI in long-term cognitive trajectories. The biological mechanism between BMI and cognitive health remains unclear, but prior research has proposed several pathways linking high BMI to greater cognitive decline [5, 38, 39]. Notably, potential biological mechanisms include systemic inflammation leading to neuroinflammation, impairments in cerebral circulation that disrupt the ability to match blood flow to the metabolic demands of neurons, and cerebrovascular dysregulation characterized by reduced functional hyperemia and endothelial dilation [5, 39, 40]. Evidence from recent randomized trials of semaglutide in patients with mild Alzheimer’s disease (AD) suggests that short-term reductions in BMI may not translate into measurable cognitive benefits [41]. Cognitive endpoints in early AD may be relatively insensitive to modest short-term changes, making small effects difficult to detect over a 2-year window [42]. Moreover, metabolic improvements may precede measurable clinical cognitive changes, indicating that longer exposure periods may be required to observe cognitive effects following risk-factor modification [43]. Thus, it is warranted to use a longer exposure duration when interpreting the relationship between BMI and cognitive outcomes. Additionally, some studies observed long-term high BMI may be associated with alterations in the gut microbiota, leading to reduced short-chain fatty acid production, which may impact brain function by modifying microglial activity and disrupting gene expression for neurons [44, 45]. Furthermore, our findings highlight the association of caBMI with memory and executive function, offering a more specific understanding of the caBMI-cognitive decline association [46, 47]. Previous research has indicated that high BMI is associated with impaired hippocampal vascular health and structural changes in regions involved in memory functions, such as the angular gyrus, which induce declines in memory processing and retrieval [48, 49]. On the other hand, executive function is regulated by the prefrontal cortex, which is responsible for complex behaviors, such as decision-making and reasoning [50]. Gray matter volume and blood flow in the prefrontal cortex may reduce as BMI increases, which may help explain the negative association between caBMI and decline in executive function [51, 52].

In this study, we innovatively identified year 8 as a critical period during which caBMI exhibited its most pronounced association with cognitive decline, highlighting an informative reference window for understanding long-term cumulative BMI in relation to cognitive trajectories. This time frame aligns with prior research on BMI trajectories, which found that changes in BMI are linked to cognitive decline approximately seven years later [53]. Besides, subgroup analyses further reveal an increased susceptibility to the association between caBMI and cognitive decline, particularly among older adults. This observation aligns with the previous studies that identify aging populations as more vulnerable to high BMI-related cognitive decline [54, 55]. Our findings highlight the potential relevance of weight management in older populations in relation to cognitive health. Although the estimated associations are modest in magnitude, they are statistically significant and consistent across multiple models, supporting the robustness of the findings. Several features of the modeling framework help contextualize these effect sizes. Specifically, caBMI is designed to capture long-term exposure and therefore smooths short-term variability in BMI, which may influence the apparent magnitude of associations compared with estimates based on cross-sectional or short-term measures [56, 57]. Moreover, cognitive decline is a multifactorial process shaped by demographic, behavioral, and health-related factors [58]. After adjustment for a comprehensive set of covariates, the association between caBMI and cognitive decline reflects its contribution within a multifactorial context and, therefore, may be modest in magnitude when expressed on an annualized scale. Finally, because cognitive change is gradual and was modeled as the annualized change in standardized cognitive scores (SD/year), numerically small coefficients may be expected, while such effects may still accumulate over long follow-up periods.

From a clinical perspective, the results of caBMI underscore the importance of longitudinal BMI monitoring and sustained weight management interventions to mitigate the cumulative impact of BMI on cognitive decline, given that cognitive risk appears to be more strongly associated with long-term cumulative BMI exposure rather than short-term BMI fluctuations. Furthermore, the identification of an 8-year period with the strongest observed association highlights the relevance of considering timing when evaluating associations between BMI trajectories and cognitive outcomes. From a policy perspective, our findings on caBMI emphasize the potential relevance for public health policies that prioritize strategies addressing the cumulative burden of obesity. Such strategies may prioritize promoting sustained lifestyle patterns that support healthy BMI trajectories. Based on the observed temporal pattern, we further highlight the importance of considering long-term BMI trajectories in cognitive health monitoring. This pattern suggests that cumulative exposure over preceding years may assist in risk stratification, with implications for the design of prevention-oriented research and public health planning.

This study includes several limitations. First, reliance on self-reported BMI may introduce recall bias and social desirability bias, which may affect measurement accuracy. Age-related height reduction may also lead to modest overestimation of BMI. In addition, BMI does not distinguish fat from lean mass or capture central adiposity; BMI measurements in older adults or individuals with depression or chronic disease may be subject to additional measurement error, and averaging cumulative BMI exposure may obscure periods of rapid weight change that may be informative. Second, the presence of missing data constrains the generalizability of our findings; the findings are primarily applicable to individuals with characteristics similar to those of the analytic sample. Analyses incorporating lag windows are widely used in dementia epidemiology [59], but different lag lengths may be subject to different sources of bias. Shorter lag periods (e.g., 2–4 years) may remain more susceptible to protopathic bias [59]. In addition, although inverse probability weighting was applied to address potential bias due to differential participation and survival, some degree of selection bias cannot be entirely excluded; therefore, findings from longer lag windows should be interpreted with caution [60, 61]. Across lag windows, effect estimates should be interpreted as reflecting the consistency of observed associations as the time gap between caBMI measurement and cognitive outcomes increases. Third, by design, this study examines associations rather than causal relationships. Although we controlled for a good number of covariates, potentially relevant covariates may not have been accounted for due to data availability, such as environmental exposures, physical activity, traumatic brain injury (TBI), and other neurological conditions, which may influence the association between caBMI and cognitive decline.

Our study has several notable strengths. First, we innovatively captured the long-term effects of BMI, offering a more nuanced understanding of its association with cognitive outcomes, compared with traditional single-point BMI measurements. By explicitly implementing multiple lag windows, our study allows for a more explicit examination of temporal sequencing between cumulative BMI exposure and subsequent cognitive outcomes. Second, our analysis identified year 8 as a key association window in which the association between cumulative BMI and cognitive decline is most pronounced. The consistency of associations across multiple lag windows supports the robustness of the observed exposure–outcome patterns under varying exposure–outcome time gaps. Third, the 24-year longitudinal design enables a comprehensive assessment of BMI trajectories and their associations with cognitive decline, providing valuable insight into long-term patterns. Using a time-updated cumulative exposure metric, our study captures long-term BMI burden more comprehensively than traditional single-point or short-term change measures. In addition, inverse probability weighting (IPW)-based sensitivity analyses yielded estimates consistent with the primary analyses, further supporting the robustness of the findings.

## Conclusions

The study highlights the significant association between greater caBMI and accelerated decline in global cognition, memory, and executive function, and identifies year 8 as a critical period with the strongest association. These findings provide evidence for monitoring long-term BMI trajectories in clinical settings and public health policies to promote cognitive health in aging populations. Further research is warranted to investigate the biological mechanisms underlying the observed associations between cumulative BMI and cognitive decline and to inform future intervention studies.

## Supplementary Information

Below is the link to the electronic supplementary material.

Supplementary file1 (DOCX 197 KB)

## Data Availability

The data used in this study were obtained from the Health and Retirement Study (HRS), conducted by the Institute for Social Research at the University of Michigan. The HRS data are publicly available and can be accessed at https://hrsdata.isr.umich.edu/data-products.
