# Supplementary material for: Association between cumulative average BMI and cognitive decline: a 24-year cohort study
Source: J Neurol. 2026 Feb 27;273(2):166. doi: 10.1007/s00415-026-13696-2 (PMC12948888; doi:10.1007/s00415-026-13696-2)
Supplement: Supplementary file 1 — Supplementary file1 (DOCX 197 KB) [file 415_2026_13696_MOESM1_ESM.docx]

**Supplementary Table 1. Baseline characteristics of participants included and excluded in the study**

| Variables | Included N=8252 | Excluded N=31878 |
| --- | --- | --- |
| Age (mean [SD] in years) | 59.0 (5.6) | 53.7 (17.9) |
| Gender (n [%]) |  |  |
| Men | 3446 (41.8) | 14053 (44.1) |
| Women | 4806 (58.2) | 17825 (55.9) |
| Race/Ethnicity (n [%]) |  |  |
| Non-Hispanic White | 6492 (78.7) | 19463 (61.2) |
| Non-Hispanic Black | 1024 (12.4) | 6487 (20.4) |
| Non-Hispanic Other | 146 (1.8) | 1367 (4.3) |
| Hispanic | 590 (7.2) | 4483 (14.1) |
| Educational Attainment (n [%]) |  |  |
| Lower Than High-school | 1548 (18.8) | 8230 (25.8) |
| GED | 418 (5.1) | 1567 (4.9) |
| High-school graduate | 2817 (34.1) | 8469 (26.6) |
| Some College | 1796 (21.8) | 7390 (23.2) |
| College and above | 1673 (20.3) | 6204 (19.5) |
| Employment Status (n [%]) |  |  |
| Employed | 726 (8.8) | 1354 (13.9) |
| Unemployed | 166 (2.0) | 65 (0.7) |
| Retired | 2796 (33.9) | 6550 (67.3) |
| Disabled | 258 (3.1) | 390 (4.0) |
| Not in labor force | 4306 (52.2) | 1380 (14.2) |
| Insurance (n [%]) |  |  |
| Yes | 7436 (90.6) | 9266 (95.6) |
| No | 773 (9.4) | 426 (4.4) |
| Smoking (n [%]) |  |  |
| No smoke | 3111 (39.5) | 4202 (44.3) |
| Ever smoke | 3117 (39.5) | 3980 (41.9) |
| Current smoker | 1659 (21.0) | 1311 (13.8) |
| Depression (n [%]) |  |  |
| Yes | 7254 (87.9) | 1595 (19.7) |
| No | 995 (12.1) | 6495 (80.3) |
| BMI (mean [SD]) | 27.3 (5.1) | 25.9 (5.0) |
| Number of Chronic Diseases (mean [SD]) | 0.8 (0.9) | 1.3 (1.1) |

Abbreviations: SD: standard deviation; BMI: Body Mass Index; GED: General Educational Development

**Supplementary Table 2. The association between cumulative average BMI and memory decline**

|  | Model 1 | | Model 2 | | Model 3 | |
| --- | --- | --- | --- | --- | --- | --- |
|  | Coefficient (95% CI) | p-value | Coefficient (95% CI) | p-value | Coefficient (95% CI) | p-value |
| caBMI | -0.0074  (-0.0411,  0.0262) | 0.665 | 0.0022  (-0.0317,  0.0360) | 0.900 | 0.0029  (-0.0316,  0.0374) | 0.869 |
| caBMI*Time | -0.0016  (-0.0021,  -0.0011) | <0.001 | -0.0018  (-0.0023,  -0.0012) | <0.001 | -0.0017  (-0.0023,  -0.0011) | <0.001 |

Note: Model 1 includes demographic covariates (age, gender, race/ethnicity). Model 2 additionally includes socioeconomic status (educational attainment, employment status, and insurance status). Model 3 further includes lifestyle and health conditions (smoking, depression, and number of chronic diseases)

Abbreviation: caBMI: Cumulative Average Body Mass Index

**Supplementary Table 3. The association between cumulative average BMI and decline in executive function**

|  | Model 1 | | Model 2 | | Model 3 | |
| --- | --- | --- | --- | --- | --- | --- |
|  | Coefficient (95% CI) | p-value | Coefficient (95% CI) | p-value | Coefficient (95% CI) | p-value |
| caBMI | 0.0043  (-0.0278,  0.0364) | 0.793 | 0.0162  (-0.0160,  0.0484) | 0.324 | 0.0223  (-0.0103,  0.0550) | 0.180 |
| caBMI*Time | -0.0029  (-0.0034,  -0.0024) | <0.001 | -0.0031  (-0.0036,  -0.0025) | <0.001 | -0.0028  (-0.0034,  -0.0021) | <0.001 |

Note: Model 1 includes demographic covariates (age, gender, race/ethnicity). Model 2 additionally includes socioeconomic status (educational attainment, employment status, and insurance status). Model 3 further includes lifestyle and health conditions (smoking, depression, and number of chronic diseases)

Abbreviation: caBMI: Cumulative Average Body Mass Index

**Supplementary Table 4. The association between cumulative average BMI and cognitive decline among participants without chronic disease at baseline**

|  | Global Cognition | | Memory | | Executive Function | |
| --- | --- | --- | --- | --- | --- | --- |
|  | Coefficient (95% CI) | p-value | Coefficient (95% CI) | p-value | Coefficient (95% CI) | p-value |
| caBMI | 0.0216  (-0.0361, 0.0792) | 0.464 | 0.0032  (-0.0537, 0.0602) | 0.912 | 0.0362  (-0.0164, 0.0888) | 0.177 |
| caBMI*Time | -0.0030  (-0.0038  -0.0021) | <0.001 | -0.0015  (-0.0024  -0.0006) | 0.001 | -0.0029  (-0.0038  -0.0021) | <0.001 |

Abbreviation: caBMI: Cumulative Average Body Mass Index

**Supplementary Table 5. The association between cumulative average BMI and cognitive decline after inverse probability weighting (IPW)**

|  | Global Cognition | | Memory | | Executive Function | |
| --- | --- | --- | --- | --- | --- | --- |
|  | Coefficient (95% CI) | p-value | Coefficient (95% CI) | p-value | Coefficient (95% CI) | p-value |
| caBMI | 0.0134  (-0.0222  0.0491) | 0.460 | 0.0033  (-0.0317  0.0384) | 0.852 | 0.0200  (-0.0131  0.0530) | 0.236 |
| caBMI*Time | -0.0030  (-0.0036  -0.0024) | <0.001 | -0.0017  (-0.0023  -0.0011) | <0.001 | -0.0028  (-0.0034  -0.0022) | <0.001 |

Abbreviation: caBMI: Cumulative Average Body Mass Index

**Supplementary Figure 1. Calculation of cumulative average BMI**


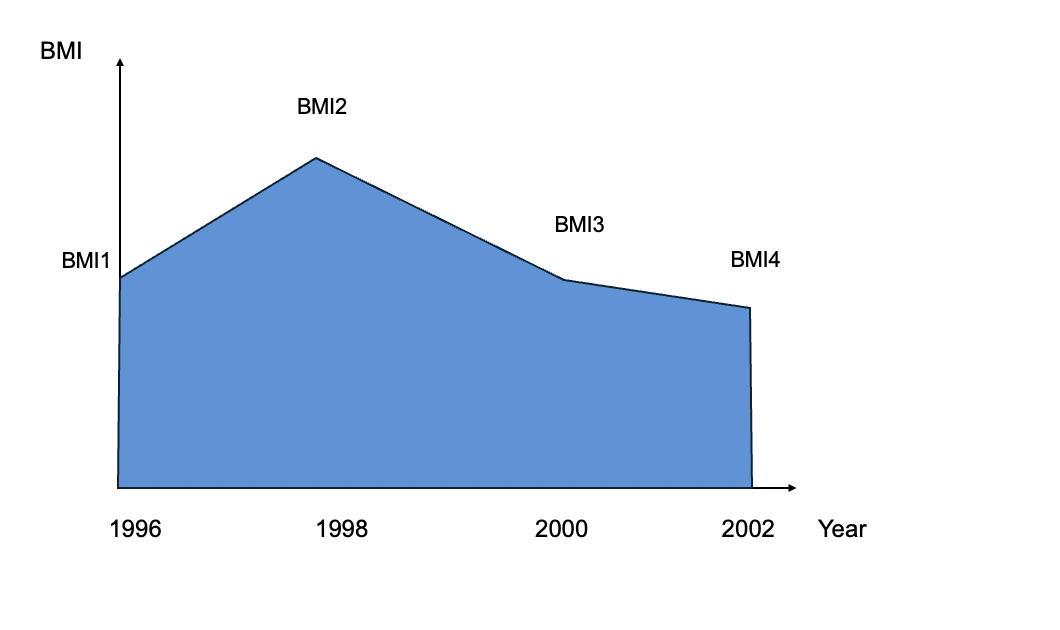


The figure illustrates the calculation of cumulative average BMI (caBMI) across multiple time points. For example, BMI values (BMI1, BMI2, BMI3, and BMI4) were recorded at different wave times (e.g., 1996, 1998, 2000, and 2002). According to the trapezoid rule, cumulative BMI (cBMI) was obtained by summing the area under curve. cBMI between 1996 to 2002 could be calculated as (BMI1+BMI2)$\times$(1998-1996)/2+ (BMI2+BMI3)$\times$(2000-1998)/2+ (BMI3+BMI4)$\times$(2002-2000)/2. The cumulative average BMI (caBMI) was then calculated by averaging cBMI values by the time intervals. caBMI for period between 1996 and 2002, a 6-year interval, was calculated by dividing the cBMI from 1996 to 2002 by the 6-year duration.
